# A clarion call to the addiction science community: It's time to resist the anti-scientific policies of the US Trump administration

**DOI:** 10.1177/14550725251325970

**Published:** 2025-04-01

**Authors:** Thomas F. Babor, Bryon Adinoff, Luke Clark, David Crockford, Zsolt Demetrovics, Paul Dietze, Jean-Sébastien Fallu, Sally Gainsbury, Gail Gilchrist, David A. Gorelick, Kathryn Graham, Derek Heim, Matilda Hellman, Anne-Marie Laslett, Caravella McCuistian, Michal Miovsky, Neo K. Morojele, Jacek Moskalewicz, Isidore S. Obot, Richard Pates, Robin Room, Marta Rychert, Aysel Sultan, Carla Treloar, Nigel E. Turner, Samantha Wells, Emily C. Williams, Katie Witkiewitz, Jason Grebely

**Affiliations:** Department of Public Health Sciences, University of Connecticut School of Medicine, Farmington, CT, USA; University of Colorado Anschutz Medical Center, Aurora, CO, USA; Department of Psychology, 8166 University of British Columbia, Vancouver, BC, Canada; Department of Psychiatry, Cumming School of Medicine, University of Calgary, Calgary, AB, Canada; Flinders University Institute for Mental Health and Wellbeing, College of Education, Psychology and Social Work 1065 , Flinders University, Bedford Park, South Australia, Australia; Institute of Psychology, ELTE Eötvös Loránd University, Budapest, Hungary; Disease Elimination Program, 104125 Burnet Institute, Melbourne, VIC, Australia; National Drug Research Institute, Curtin University, Perth, WA, Australia; School of Psychoeducation, University of Montreal, Montreal, QC, Canada; School of Psychology, University of Sydney, Sydney, NSW, Australia; Addictions Healthcare Research, Institute of Psychiatry, Psychology & Neuroscience, 4616 King's College, London, UK; Psychiatry, 12264 University of Maryland School of Medicine, Baltimore, MD, USA; Centre for Addition and Mental Health, Canada; 274071 Dalla Lana School of Public Health, University of Toronto, Toronto, ON, Canada; Psychology, 6249 Edge Hill University, Ormskirk, Lancashire, UK; Department of Sociology, Uppsala University, 751 26 Uppsala, Sweden; Faculty of Social Sciences, 3835 University of Helsinki, 00014 Helsinki, Finland; Centre for Alcohol Policy Research, 2080 La Trobe University, Melbourne, VIC, Australia; Department of Psychiatry and Behavioral Sciences, 8785 University of California, San Francisco, CA, USA; Department of Addictology, First Faculty of Medicine, 37740 Charles University, Prague, Czech Republic; Department of Psychology, 61799 University of Johannesburg, Johannesburg, South Africa; 49794 Institute of Psychiatry and Neurology, Warsaw, Poland; 570591 Centre for Research and Information on Substance Abuse, Uyo, Nigeria; 8709 University of Worcester, Worcester, UK; Centre for Alcohol Policy Research, 2080 La Trobe University, Melbourne, VIC, Australia; SHORE & Whariki Research Centre, 385563 College of Health, Massey University, Auckland, New Zealand; Department of Science, Technology and Society, 9184 Technical University of Munich, Munich, Germany; Centre for Social Research in Health, 7800 University of New South Wales, NSW, Australia; 7978 Institute for Mental Health Policy Research and Campbell Family Mental Health Research, Centre for Addiction and Mental Health, Toronto, ON, Canada; Institute for Mental Health Policy Research, Centre for Addition and Mental Health, Toronto, ON, Canada; Dalla Lana School of Public Health, University of Toronto, ON, Canada; Department of Health Systems & Population Health, 7284 University of Washington, Seattle, WA, USA; Department of Psychology, University of New Mexico, Albuquerque, NM, USA; Hepatitis and Drug Use Research Group, Kirby Institute, Australia

**Keywords:** Addiction science, addiction science editors, autonomous science, science publication, scientific integrity, addiction science

As a group of 28 addiction journal editors from 12 countries, we are urgently drawing our readers’ attention to the abrupt and drastic changes in science policy now being enacted by the current US government. We are issuing a clarion call to the addiction science community to reverse the unethical, illegal and unscientific activities of the Trump Administration and, by analogy, the activities of other governments that interfere with the pursuit of scientific knowledge to manage addiction-related problems (Balfe, 2023; Hall et al., 2012). There are three reasons for this call to action.

First, the Administration is attempting to censor scientific discourse within peer-reviewed publications. Following President Trump's Executive Order on “Defending Women from Gender Ideology Extremism and Restoring Biological Truth”, the US Centers for Disease Control and Prevention (CDC) mandated that all scientific manuscripts authored by CDC personnel and currently undergoing peer review be withdrawn so that certain “forbidden terms” relating to gender can be removed (Clark & Abbasi, 2025; Heidt, 2025; Mandavilli, 2025). The terms include “gender”, “transgender”, “pregnant person”, “transsexual” or “non-binary”. This is clearly unethical (removing authors who contributed to a publication), possibly illegal (changing an article after copyright has been transferred to a journal) and definitely unscientific (editors should not publish articles that fail to accurately describe sample characteristics in terms of sex, gender and sexual minority composition, where relevant). Censorship by the Administration runs counter to long-standing efforts by the International Society of Addiction Journal Editors (ISAJE) to promote accurate and complete reporting of sex and gender information in scientific research (Heidari et al., 2016) and to improve gender representation across member journals (Babor et al., 2023). Even worse, CDC and other government agencies have removed from public view epidemiological datasets related to a range of health topics, including the Youth Risk Behavior Survey, without explanation or justification (Clark & Abbasi, 2025; Cox et al., 2025).

Second, funding and human resources for scientific research are being arbitrarily reduced, without any viable justification in terms of improved efficiency or public policy. There are numerous reports (e.g., Gwynne, 2025) of frozen training positions, use of government databases to identify people who have worked on diversity issues, funding freezes on research and training grants, and mass firings of career employees in critical health areas. At this writing, the US Agency for International Development (US AID) is being threatened with complete elimination. Clinical trials investigating HIV treatments have been “paused”, abruptly leaving patients without lifesaving treatment and risking the development of medication-resistant viral strains (Farmer, 2025).

Third, consideration is being given to reducing the National Institutes of Health (NIH) budget and converting NIH grant funding into block grants to the states to fund research at their discretion (Severino, 2023). This is likely to result in reduced funding for marginalized and vulnerable populations. Will states’ total funding for research on alcohol, tobacco, illegal drugs and behavioral addictions match the current budgets of the National Institute on Drug Abuse (NIDA), the National Institute on Alcohol Abuse and Alcoholism (NIAAA) and other agencies? NIH has already announced a reduction in the indirect (overhead) cost rates paid to grant-receiving institutions by as much as 4 billion dollars this year (Milman, 2025).

## What can be done?

The response to these dramatic changes in US science policy must be comprehensive, collaborative and led by the major addiction science organizations. The current worldwide infrastructure of this field, perhaps as much as half of it concentrated in the USA, includes numerous federal funding sources, more than 90 specialized scholarly journals, scores of professional societies, over 200 research centers, more than 80 specialty training programs and thousands of scientists (Babor et al., 2017). Below, we describe an initial plan of action that engages all parts of the addiction science field.

## Addiction journals

- The ISAJE Board should notify the CDC and the NIH that sound journal policies and scientific writing in our field dictate that terminology relating to sex, gender and sexual minority issues are consistent, interpretable and scientifically appropriate. The terminology for various contexts and realities is constantly assessed and discussed within scientific communities, and these discussions are integral to high-quality scientific processes. Because accurate and legitimate descriptions of study samples’ sex and gender characteristics are a basic requirement in all human research, the publishers of addiction journals are encouraged to resist unjustified and unscientific efforts to interfere with them.
- ISAJE should also insist on adherence to authorship policies that disallow the arbitrary removal of US authors from manuscripts with “forbidden” terminology.

## Research societies

- The addiction field's major research societies should facilitate member petitions, position statements and editorials in their affiliated journals, re-affirming support for well-substantiated scientific terminology, diversity policies and funding for addiction research and research training.
- Annual meeting organizers should set programing agendas to take into account current threats to addiction research and treatment.
- US addiction research organizations should inform the US Congressional leadership about the practical implications of the illegal, unethical and unscientific actions described in this editorial. They should also reaffirm their support for NIAAA, NIDA, CDC and other federal agencies that fund addiction research and training both nationally and internationally.
- Research societies should make concrete efforts to communicate broadly about the societal benefits of addiction science, which include basic research on the nature of addiction, clinical studies of the most effective treatments and policy research that demonstrates how to reduce the enormous costs of alcohol, tobacco and illegal drugs, as well as behavioral addictions associated with gambling, gaming, and problematic use of the internet.

## Individual scientists

- Addiction scientists are encouraged to exercise the right of free expression in scientific publications, insisting on the continued application of the peer review system for grant funding and research publications that is independent of political interference.
- The scientific community is encouraged to speak up as citizens who have expertise in addiction science. They should describe the implications of the US Administration's executive mandates for addiction research and public health, both nationally and internationally.
- Investigators, regardless of career stage and seniority, on US federal grants need to push the leadership of research societies and the addiction journals to articulate their concerns, particularly with respect to research training and diversity issues.

## Conclusions

Common strategies and coordinated efforts are needed to protect the scientific infrastructure that has been developed over the past 50 years in the USA and worldwide. We suggest the formation of a broadly representative Coordinating Committee consisting of the many stakeholders in the addiction science field. The Committee can be charged with the development of a resistance strategy designed to prevent the further dismantling of the global engine of addiction research that has made the USA a world leader in the scientific understanding of addiction problems. The emerging intrusions into our scientific work by political and ideological operatives is not acceptable and it will make it more difficult and expensive to deal with these complex human problems in the future. The global addiction science community needs to act collectively to preserve the promise of scientific inquiry wherever it is threatened.
